# Recent progress in mass spectrometry-based urinary proteomics

**DOI:** 10.1186/s12014-024-09462-z

**Published:** 2024-02-22

**Authors:** Neha Joshi, Kishore Garapati, Vivek Ghose, Richard K. Kandasamy, Akhilesh Pandey

**Affiliations:** 1 Manipal Academy of Higher Education (MAHE), Manipal, 576104 India; 2 Institute of Bioinformatics, International Technology Park, Bangalore, 560066 India; 3 Department of Laboratory Medicine and Pathology, Mayo Clinic, 200 First Street SW, Rochester, MN 55905 USA; 4 Department of Quantitative Health Sciences, Mayo Clinic, Rochester, MN 55905 USA; 5 Center for Individualized Medicine, Mayo Clinic, Rochester, MN 55905 USA

## Abstract

Serum or plasma is frequently utilized in biomedical research; however, its application is impeded by the requirement for invasive sample collection. The non-invasive nature of urine collection makes it an attractive alternative for disease characterization and biomarker discovery. Mass spectrometry-based protein profiling of urine has led to the discovery of several disease-associated biomarkers. Proteomic analysis of urine has not only been applied to disorders of the kidney and urinary bladder but also to conditions affecting distant organs because proteins excreted in the urine originate from multiple organs. This review provides a progress update on urinary proteomics carried out over the past decade. Studies summarized in this review have expanded the catalog of proteins detected in the urine in a variety of clinical conditions. The wide range of applications of urine analysis—from characterizing diseases to discovering predictive, diagnostic and prognostic markers—continues to drive investigations of the urinary proteome.

Blood and its cellular constituents (e.g., lymphocytes) are the most commonly used specimens for laboratory testing and diagnosis. However, collecting blood samples is an invasive procedure and the development of more non-invasive methods of clinical testing and diagnosis is important for healthcare delivery [1]. The non-invasive nature of urine collection and its deployment for research as well as clinical testing offers a distinct advantage over other body fluids or tissue specimens. A simple urinalysis is often an essential component of managing different disorders including diabetes, kidney diseases and urinary tract infection [2, 3]. Notably, detecting the presence of human chorionic gonadotropin (hCG) hormone in urine to detect pregnancy is perhaps the most popular point-of-care test in medical diagnostics.

In contrast to serum or blood, which are commonly used but require invasive methods for collection, urine can be collected in a simple and non-invasive fashion and in significant quantities [4, 5]. Indeed, urine has also become a valuable specimen in the field of biomedical research due to its widespread availability among patients and the straightforward collection process. Various urine collection methods such as first-morning, 24-h or random spot collection can facilitate easy follow-up and time-based studies [6–8]. From an analytical standpoint, the composition of urine proteome is less complex than that of serum or plasma making it more feasible for proteomic analyses where abundant proteins can obscure signals derived from less abundant proteins [9, 10]. The glomeruli of a healthy kidney filter 150–180 L of urine each day of which 99% is reabsorbed [4]. Among the proteins observed in urine of healthy individuals, 70% originate in the urinary tract, while the remaining are proteins filtered from plasma [11]. Liver and kidney are major organs that maintain homeostasis in blood and urine reflects any alterations. It should be noted that the earliest described urinary biomarkers are from the field of nephrology [12, 13]. Thus, analysis of urinary proteins as biomarkers is of great importance not only for diseases of the urinary tract, but also for diseases affecting more distant organs [14]. Several analytical techniques have been employed to study urine proteome; however, mass spectrometry has emerged as a sensitive method for global urinary protein analysis. One of the earliest mass spectrometry-based proteomics analysis of urine identified 124 proteins [15] while current studies using high resolution mass spectrometry routinely identify between 1000 and 3000 proteins depending on the methods and instrumentation [10, 16].

Numerous factors such as sample collection, protein normalization, intra- and inter-individual differences in protein amount are to be considered when carrying out urine proteomics. Although urine is a less complex biospecimen, these inter- and intra-individual variations leads to the disparity in the protein content of urine. Moreover, urine collected at different times during the course of a day can also exhibit variation in the protein content. Usually, the second urine in the morning or 24-h urine is considered to be a “gold standard” for proteome analysis. In 24-h collection, the average information of urinary proteins excreted in a day can be obtained while a morning urine (usually second urine) collection avoids the diurnal variation if samples were collected at different time-points during the day [17, 18]. However, the 24-h collection of urine might not be practical due to its dependence of patient compliance and errors that often arise during collection [19]. Other factors such as protein intake, posture, circadian rhythm and normal physical activities can also affect the proteome [20, 21]. First voided or early morning (second) or spot (random) collection have been deployed to minimize the errors caused during 24-h collection [22, 23]. In such cases, the protein amount is usually normalized with respect to total creatinine by considering the protein/creatinine ratio, which is a current practice for quantifying urinary proteins [24, 25].

## Analytical methods for urinary proteomics

Urine proteome has been studied using several analytical techniques that can be unbiased, e.g., mass spectrometry-based approaches or biased, e.g. proximity extension assay-based (Olink) or aptamer based (Somalogic) approaches [26–29]. In this review, we will restrict ourselves to mass-spectrometry-based approaches that have been employed to profile proteins that make up the urine proteome in both healthy and disease states.

1. Matrix-assisted laser desorption ionization- time-of-flight mass spectrometry (MALDI-TOF–MS): MALDI-TOF mass spectrometry has also been used in the study of urine proteomics [30, 31]. This approach makes use of protein or peptide profiling in samples that have been crystallized using a MALDI matrix, often 2,5-Dihydroxybenzoic acid. MALDI is a soft-ionization technique that can analyze hundreds of samples or analytes in a short period of time. MALDI-TOF/TOF-based analyses permit accurate identification of peptides from crude mixtures such as the urinary proteome.
2. Capillary electrophoresis coupled with mass spectrometry (CE-MS): This method is based on use of a mass spectrometer with capillary electrophoresis (CE) at the front end [32, 33]. Based on how proteins move across a gel when subjected to an electrical field, CE separates proteins in a single step with excellent resolution. CE-MS offers the following benefits: 1. It provides fast separation [34]; 2. It is robust and uses cost-effective capillaries rather than costly LC columns [35]; 3. It is compatible with a broad range of buffers and analytes [36] and 4. It provides a stable constant flow, without the need for elution gradients that might otherwise interfere with MS detection [37]. As high molecular weight proteins tend to precipitate at low pH used for CE, analysis of such proteins with CE is not efficient. In addition, the volume of sample that can be introduced into a capillary for separation by CE is small, potentially limiting the detection sensitivity of CE-MS [38–40]. Despite efficient separation and low sample requirements, CE-MS alone cannot provide definitive identification (sequence of peptides and proteins) being analyzed. Identification of peptides by their molecular weight alone can lead to false positives. For confident identification and quantification, fragmentation of the precursor ions followed by detection of fragment ions is carried out using tandem mass spectrometry (MS/MS) approaches. Studies have been reported in which CE-MS is performed in parallel with LC–MS/MS to map the precursor ions identified in CE-MS data to peptide sequences using fragmentation information in LC–MS/MS data, which can be a tedious process [41, 42]. However, several studies have utilized ESI for ionization of analytes that are separated using CE prior to performing MS/MS. Such analyses, termed CE-ESI–MS/MS, also allow for definitive peptide identification [43–45]. However, it is important to note that CE-ESI–MS/MS favors basic and hydrophilic peptides with low molecular masses, thus leading to an underrepresentation of peptides with relatively larger molecular weight [45].
3. Liquid chromatography coupled with tandem mass spectrometry (LC–MS/MS): The majority of LC–MS-based methods involve trypsin digestion of proteins followed by LC-based separation of the resulting tryptic peptides prior to tandem mass spectrometry [10, 46]. This technique has been applied to identify and quantify thousands of urine proteins including low abundance proteins [10, 16, 47]. One of the major advantages of this approach is the high resolution of separation of the proteins by LC columns based upon different properties of the peptides including hydrophobicity, size or affinity prior to mass spectrometry analysis. This separation of the peptides provides an excellent coverage of proteins identifying the high as well as low abundance proteins in urine [48]. Moreover, this technique can be automated and used to separate large amounts of analytes (protein/peptides) on an high performance liquid chromatography (HPLC) column or tiny amounts of analytes (peptides) on a capillary LC column [49]. Despite providing high-resolution separation, this method is sensitive to interfering compounds in the urine (e.g., salts), which have to be removed during sample processing. The time taken for data acquisition makes it somewhat challenging to analyze very larger sample sets [40].

## Urinary proteomics

**Fig. 1:**
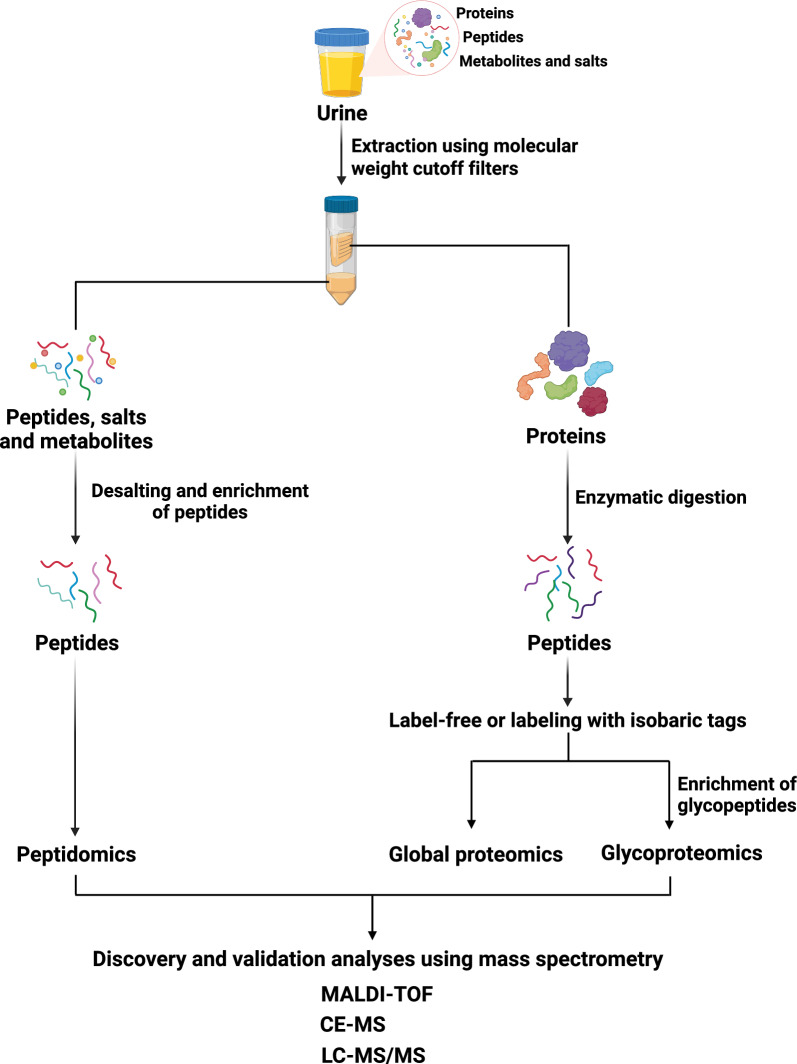
A schematic of the various analytical approaches for urine proteomics. The workflow of mass spectrometry-based approaches to enrich and study urinary proteins, glycoproteins and peptides is shown

**Fig. 2:**
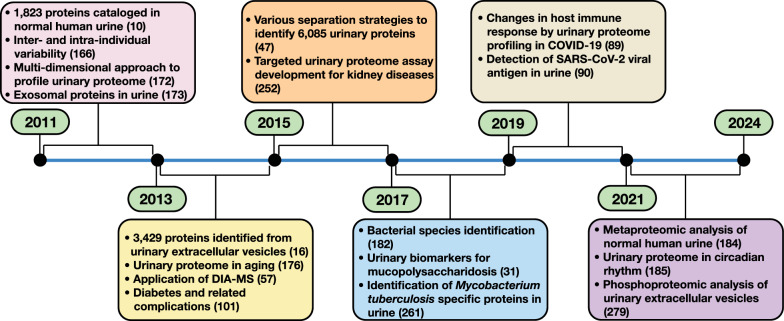
A timeline of key studies in the field of urinary proteomics: Highlights of major studies and applications of urinary proteomics over the past decade

Urinary proteomics is a burgeoning field of research that focuses on the comprehensive analysis of proteins present in urine (Fig. 1) providing valuable insights into various physiological and pathological processes. Due to its potential in the early diagnosis, prognosis and monitoring of a wide range of diseases non-invasively, urinary proteomics has gained prominence in biomarker discovery. Through advancements in mass spectrometry and high-throughput technologies, unraveling the intricate proteomic signatures within urine has now become feasible, shedding light on the normal [10, 16] as well as disease mechanisms and facilitating personalized medicine in several diseases such as urothelial cancer [50], prostate cancer [51], diabetes [52] and chronic kidney disease (CKD) [53]. Numerous mass spectrometry-based proteomics studies have been performed to profile and quantify the urinary proteins (Table 1). In mass spectrometry-based urine proteome investigations, LC–MS/MS is the most often used technology [10, 54]. More recently, a number of targeted mass spectrometry approaches as well as data dependent acquisition (DDA) [55] and data independent acquisition (DIA) [56, 57] approaches have been used as quantification techniques in urine proteomics studies. Specific biomarker-based investigations for urine proteomics have used targeted mass spectrometry methods such as multiple reaction monitoring (MRM) [58, 59] parallel reaction monitoring (PRM) [60, 61] and a newly developed SureQuant approach for absolute quantification and validation of the targeted proteins [62]. Following sections provide a brief overview of cataloging studies or interesting applications ranging from normal proteome profiling to biomarker discovery for numerous disorders including cancer that were reported over the past decade. A pictorial representation for the same is depicted in Fig. 2.

**Table 1:** A list of major urinary proteomic studies using mass-spectrometry since 2011

Condition	Salient features	Technology used	References
Normal human urinary proteomics	Studies involved cataloging, quantifying and profiling proteins in normal human urine as well as urine protein changes associated with, pregnancy, normal renal function, tobacco smoking and individual variation	LC–MS/MS HPLC-Chip-MS/MS	[10] [166] [170] [171] [172] [173] [174] [175] [176] [177] [178] [57] [16] [179] [180] [142] [47] [181] [182] [183] [184] [185]
Urine proteomics in cancer	Application of urinary proteomics in identification of non-invasive diagnostic biomarkers for numerous cancer types including bladder cancer, prostate cancer, lung cancer, gastric cancer and cholangiocarcinoma	MALD-TOF LC–MS/MS CE-MS	[70] [186] [75] [76] [187] [72] [188] [73] [189] [190] [191] [192] [193] [194] [195] [196] [197] [198] [199] [200] [74] [201] [151] [202] [203] [204] [205] [206] [207] [208] [209] [66] [210] [211] [212] [213] [214] [215] [216] [217] [218] [219] [220] [221] [222] [223] [224] [62] [225] [226]
Urine proteomics in other pathologies	Pathological conditions such as urinary tract infections, diabetic nephropathy, chronic kidney disease, coronory artery disease, pre-eclampsia, tuberculosis, SARS-CoV-2 and other immunological morbidities have been assessed with the help of urine proteomics	SELDI-TOF CE-MS LC–MS/MS Targeted mass spectrometry	[227] [228] [229] [230] [231] [232] [233] [234] [235] [236] [237] [101] [85] [238] [239] [240] [241] [242] [243] [244] [245] [246] [247] [248] [249] [250] [251] [252] [253] [254] [255] [99] [256] [257] [258] [259] [260] [105] [261] [262] [263] [31] [264] [265] [266] [267] [268] [269] [90] [6] [270] [271] [91] [272] [273] [274] [56] [275] [276] [277] [278] [87] [279] [280] [281] [282] [283] [284]

### Cataloging studies

A number of studies aiming to profile urinary proteins in apparently healthy individuals have contributed to our understanding of normal human urinary proteins and established the baseline for urinary proteomics in several other disorders. Profiling the proteome of urine using mass spectrometry started in the early 2000s. The first LC–MS/MS analysis reported 124 proteins in the normal human urine [15]. Although this work was not expressly intended to identify biomarkers, it did demonstrate the untapped potential of urinary proteome and its usefulness for biomarker discovery. Subsequently, a proteomic study of normal human urine profiled 226 proteins separated using three different approaches. This study employed three different methods of protein and peptide separation in parallel, i.e., GeLC-MS/MS, LC–MS/MS and 2D—LC–MS/MS to achieve such depth [63] with more than 100 proteins identified, including proteins with low molecular mass. Additionally, 171 urinary proteins were newly identified along with 4 male-specific proteins in this study [63]. Shortly thereafter, an LC–MS/MS study was performed identifying more than 1500 proteins in human urine [64]. A proteomic analysis of non-prefractionated urine samples revealed around 1300 proteins including phosphoproteins in the normal human urine proteome for the first time demonstrating the utility of mass spectrometry-based proteomics to identify proteins that are post-translationally modified [65]. In 2011, in a study by Marimuthu et al. 1823 proteins were detected in a comprehensive analysis of human urine proteome [10] identifying around 600 proteins which were not reported to constitute human urine proteome. Many more studies have subsequently been carried out focussing on expanding the protein catalog of urinary proteome. A combination of different analytical approaches to increase the sensitivity and reproducibility of human urine proteome analysis by mass spectrometry yielded 3429 proteins [16]. By utilizing various separation strategies, including direct one-dimensional liquid chromatography-tandem mass spectrometry (LC–MS/MS), two-dimensional LC–MS/MS and gel-eluted liquid fraction entrapment electrophoresis/liquid-phase isoelectric focusing followed by two-dimensional LC–MS/MS, a total of 6085 proteins were found in the human urine proteome representing the largest database of the urine proteome till date [47].

### Urinary proteomics in bladder cancer

Proteomics of urine has been widely adopted in multiple diseases especially in cancer for biomarker discovery in a non-invasive fashion. Although urine proteomics has often been performed in the context of renal disorders, some cancers such as lung [66] and prostate [67] cancer have been studied using urine proteomics approach. Over the past decade, nearly 400 studies have been carried out in cancer patients using urine samples. In bladder cancer, the urine is in direct contact with the tumor and its analysis often yields key proteomic alterations [12]. Some of the studies performed on bladder cancer urine proteomics since 2011 are discussed below.

Selevsek et al. reported a mass spectrometry-based targeted proteomics approach to quantify urinary proteins and peptides in bladder cancer. Stable isotope-labeled peptides were used for absolute quantification of nanogram amounts of endogenous peptides in urine using selected reaction monitoring (SRM) [68]. This study showed the potential of targeted mass spectrometry approaches in detection of peptides and proteins in the urine. An increased level of alpha-1-antitrypsin (A1AT) glycoprotein in urine from bladder cancer cohort was identified in a LC–MS/MS-based urine proteomics [69]. In this study, ~ 200 urinary glycoproteins were enriched and subsequently analyzed using a label-free quantitative approach for identification of novel non-invasive biomarkers to diagnose bladder cancer. An overexpression of ADAM28 protein in urine was found to be associated with bladder cancer invasion by two different groups [70, 71]. A combination of different proteomic approaches including western blot and LC–MS/MS was reported in these studies. Increased levels of several other proteins such as apolipoprotein E, alpha-1-antitrypsin and fibrinogen were also found and believed to be associated with recurrence in an LC–MS/MS analysis of urine from non-muscle invasive bladder cancer [72]. A phosphoproteomic study showed a downregulation of a profilin-1 in urine suggesting its association with bladder cancer invasion [73]. This study applied immobilized metal affinity chromatography (IMAC)-based phosphopeptide enrichment to urine samples. A high-resolution mass spectrometry-based analysis revealed a multiplex protein panel of five urine proteins including coronin-1A, apolipoprotein A4, semenogelin-2, gamma synuclein and DJ-1/PARK7 that could serve as diagnostic biomarkers in transitional bladder cancer [74]. Apart from discovery-style proteomics, numerous targeted mass spectrometry approaches have been deployed to validate the protein expression in urine from bladder cancer patients [75, 76]. Not only secreted proteins but extracellular vesicles in urine could also be effectively used as a sample for bladder cancer biomarker discovery [77–81]. Extracellular vesicles are secreted by the cells from various tissues into biological fluids and have roles in intercellular communication and signaling [82] and hence could be used in biomarker research. Recently, Carvalho et al. reported proteomic signatures that can indicate recurrence of bladder cancer [83]. LC–MS/MS-based monitoring of urine proteome at different stages of the disease have shown the utility of these proteins to be used as biomarkers for recurrence of bladder cancer [83]. These studies prove the ability of urine proteomics in discovering non-invasive biomarkers for bladder cancer and could be applied to other cancers.

### Urinary proteomics in diabetic nephropathy

In addition to cancer, urinary proteomics has been used to characterize a number of other illnesses, including rheumatoid arthritis [84, 85], urinary tract infections [86], mucopolysaccharidosis [31], COVID-19 [87–90], neurological diseases [91–94] and diabetes [95]. A discovery study of urine from type-1 diabetes patients resulted in the identification of proteins that are dysregulated in type-1 diabetes and understanding the molecular mechanisms associated with the complications in type-1 diabetes [96]. According to a study by Fisher et al., patients with diabetic nephropathy had lower levels of urine proteins (e.g., uteroglobin) that are linked to the glomerular filtration rate. This was discovered using immunodepletion of high abundance urinary proteins, followed by fractionation and MALDI-MS analysis [97]. In 2013, Manwaring et al. showed how targeted mass spectrometry-based proteomics might be used to find new diagnostic biomarkers in pediatric patients with Fabry disease and type-1 diabetes [98]. Several other studies have been conducted to elucidate the proteomic alterations in urine from diabetes and associated complications such as nephropathy and retinopathy making it feasible to identify biomarkers for these conditions [99–101]. Lysosomal proteins were detected abundantly in the urine from young individuals with type-1 diabetes suggesting its correlation with inflammation in the kidney [102]. A similar study by another group showed the variations in lysosomal function are associated with alterations at proteome level in urine from type-1 diabetes [103]. In another study, alterations in the kallikrein-kinin system were shown to be involved in type-1 diabetes [104]. Urinary chronic kidney disease 3 (CKD3) classifier may be used to predict mortality in people with type 2 diabetes and microalbuminuria as well as to serve as a progressive marker for diabetic nephropathy [105]. Potential prospects for use in pathology and treatment include elevated levels of vascular cell adhesion molecule 1 (VCAM-1) and neprilysin in patients with baseline diabetic nephropathy and diabetic nephropathy-treated patients with persisting albuminuria, respectively [106]. Recently, a data independent analysis-mass spectrometry (DIA-MS) approach was applied to the urinary exosomal proteomics to uncover non-invasive protein-based urinary biomarkers in diagnosing diabetic nephropathy [107].

## Urine glycoproteomics

A large number of proteins excreted in the urine are glycoproteins with either N- and/or O-linked glycosylation and may be of potential diagnostic value [108, 109]. Prostate specific antigen (PSA) is an N-linked glycoprotein with a key role in the diagnosis of prostate cancer. Urinary levels of PSA have been shown to be valuable in the differential diagnosis of prostate cancer and benign prostatic hyperplasia when serum levels of PSA are inconclusive [110]. A number of studies have reported on the N-linked glycoproteome of urine and urinary exosomes (Table 2). Methods and technologies used for analyzing the urinary glycoproteome have evolved over the years for characterization at the level of released glycans, deglycosylated peptides and intact glycopeptides with site-specific glycosylation information. For instance, Blaschke et al. have characterized n-glycans released enzymatically from urinary proteins along with prostatic secretions by using MALDI-imaging mass spectrometry [108]. They report that a majority of n-glycans from urinary glycoproteins are biantennary, fucosylated and sialylated [108]. However, the identification of glycoproteins and their glycosylation sites requires alternate methods of sample preparation and mass spectrometry analysis. Huo et al. reported a method to identify soluble urinary glycoproteins and glycoproteins from urinary extracellular vesicles (EVs) along with their glycosylation sites for potential applications towards comprehensive screening [111]. They enriched glycopeptides using hydrophilic interaction liquid chromatography (HILIC) and enzymatically deglycosylated them using PNGase F to identify over 600 n-glycoprotein groups in urine. However, deglycosylated peptide analysis does not provide information on the composition of glycans attached to the identified site, which is critical for biomarker discovery and screening. This information can be obtained by analyzing intact glycopeptides. Halim et al. captured urine-derived glycoproteins containing sialic acid using hydrazide chemistry, digested the captured proteins and analyzed the attached glycopeptides after releasing them by desialylation [54]. LC–MS/MS-based analysis of intact glycopeptides from complex samples has evolved considerably over the past few years [112]. Saraswat et al. enriched glycopeptides derived from urinary exosomes using both lectin affinity and size exclusion chromatography methods and analyzed intact n-glycopeptides by LC–MS/MS [113]. Belczacka et al. performed MS analysis using different technologies on intact urinary glycopeptides to identify both N- and O-linked glycopeptides [114].

**Table 2:** A list of mass spectrometry-based glycoproteomic studies in urine since 2011

Condition	Salient features	Technologies used	References
Normal human urinary glycoproteomics	Glycoproteomic analysis of normal human urine in order to profile site-specific N- and O-glycosylation sites in the urine as well as urinary exosomes. Application of liquid handling system to enrich N-linked glycopeptides from urine was also demonstrated	LC–MS/MS CID-MS/MS LC–MS/MS along with Western blot	[54] [113] [109] [111] [114] [285] [286] [287]
Cancer	Differential urinary glycoproteomic analysis to evaluate the alterations in different cancers (prostate cancer, papillary thyroid carcinoma, bladder cancer and hepatocellular carcinoma) at glycoprotein levels that could serve as biomarker or potential therapeutic targets	LC–MS/MS PRM-MS/MS	[288] [289] [290] [115] [61] [116] [117]
Other diseases	Human urine glycoproteomic analysis in kidney-related diseases, diabetic nephropathy and Parkinson's disease	MALDI-TOF/TOF 2D-LC–MS/MS LC–MS/MS	[291] [292] [293]

Some of the above-mentioned technologies have been applied to study urinary glycoproteomic alterations in various cancers and disorders of the urinary tract and other organ systems. Sathe et al. profiled the urinary n-glycoproteome by analysis of subtypes of bladder cancer by quantitative analysis of deglycosylated n-glycopeptides to identify altered glycosylation levels between the subtypes [115]. Wang et al. performed an exploratory study of enzymatically deglycosylated peptides from prostate cancer patient urine-derived proteins to identify 1044 n-glycosylation sites [116]. Dong et al. developed targeted assays for a three-signature panel of urinary glycoproteins relevant to prostate cancer. They developed a PRM assay to detect peptides from urinary proteins after enzymatic deglycosylation [61]. More recently, Li et al. profiled intact N-linked glycopeptides from urinary extracellular vesicle (EV)-derived glycoproteins in hepatocellular carcinoma (HCC). They identified 756 intact glycopeptides with quantitative information and describe significant changes in the glycoproteome of urinary EVs in HCC [117]. The potential for quantitative analysis of N- and O-linked glycopeptides from urinary glycoproteins is immense in areas of disease characterization and biomarker development. However, it should be noted that available assays for measuring n-glycans and n-glycopeptides in urine can be laborious with multiple sample processing steps [108]. Therefore, there is also a need to optimize these methods to simpler formats before large-scale deployment as diagnostic markers.

## Urinary peptidomics

Peptidomics is an emerging field of study that focuses on the comprehensive characterization and quantification of endogenous generated peptides in biological samples including tissues, body fluids and cells. These endogenous peptides play crucial roles in diverse biological and metabolic processes such as communication and signaling, immune response and enzymatic regulation by serving as peptide hormones [118], neuropeptides [119], cytokines [120] and enzyme inhibitors [121]. Numerous applications of peptidomics have led to its adoption across multiple research areas including clinical diagnostics, biomarker discovery and pharmacology. Researchers employ various analytical techniques such as mass spectrometry, liquid chromatography and electrophoresis to study the peptidome from complex biological matrices [122–124]. Within the realm of human peptidomics, numerous investigations have been carried out to explore the peptidome of different tissues and diseases. For instance, in a study by Nongonierma and Fitzgerald, the authors characterized peptides derived from dietary proteins to determine their inhibitory effects on dipeptidyl peptidase IV in the human gastrointestinal tract. Their findings highlighted the potential of dietary protein-derived peptides as inhibitors of dipeptidyl peptidase IV with potential implications for managing conditions like diabetes [125]. Another study by Li et al. delved into the peptidomics of human infantile hemangioma tissue. Using LC–MS/MS, the researchers identified and compared endogenous peptides in normal skin and hemangioma tissue. This study provides insights into the role of endogenous peptides in hemangioma development and may contribute to the development of targeted treatments for this condition [126]. Notably, peptidomics has been carried out on several body fluids such as urine, serum [127, 128] and cerebrospinal fluid (CSF) [129, 130] where such endogenously generated peptides are likely to be abundant.

Urinary peptidomics focuses on the study of peptides that can be detected in urine (Fig. 1). Urine peptidomics has gained significant attention in recent years due to its potential for biomarker discovery and disease diagnosis [4, 131–133]. Studies have been conducted to explore the peptidome of urine and its clinical applications in cancer [134] and numerous other disorders including chronic kidney disease [135], celiac disease [136] and diabetic nephropathy [137]. Table 3 provides a brief list of notable applications of urinary peptidomics in various physiological conditions. Various facets of urinary peptidomics under both normal and diseased conditions, including cancer, are elaborated upon in the following sections.

**Table 3:** A list of mass spectrometry-based urinary peptidomics studies in cancer and other diseases since 2011

Condition	Salient features	Technology used	References
Normal human urine peptidomics	Numerous separation and extraction method developed for efficient enrichment of endogenous urinary peptides in healthy individuals. Along with profiling, age-related urinary peptidomics has been carried out in individuals with different age groups. These studies provide substantial foundation for disease-related peptide identification in urine	CE-TOF–MS nanoLC-TOF–MS MALDI-TOF–MS LC–MS/MS Q-TOF LC–MS	[138] [294] [139] [145] [146] [142] [144] [41] [143] [295] [296]
Urine peptidomics in cancer	Differential urinary peptidomic analysis for several cancer types including bladder cancer, ovarian cancer, prostate cancer and renal cell carcinoma (RCC) has been performed for detecting non-invasive peptide biomarkers in urine for clinical manifestation and disease management	MALDI-TO-MS LC–MS/MS CE-MS PRM-MS	[297] [298] [299] [150] [151] [300] [153] [134]
Urine peptidomics in other diseases	Non-invasive biosignature identification in diverse conditions including chronic kidney diseases (CKD), in infection, celiac disease, diabetes, hypertension, neurological disorders, in kidney transplant and drug monitoring, COVID-19, preeclampsia and cardiovascular disorders using mass spectrometry-based urine peptidomics in both discovery and targeted fashion	MALDI-TOF/TOF MRM-MS LC–MS/MS CE-MS Q-TOF LC–MS	[301] [42] [302] [156] [303] [304] [137] [305] [306] [307] [308] [154] [159] [309] [158] [310] [311] [312] [313] [160] [157] [314] [136] [161] [315] [316] [162] [317] [318]

### Cataloging studies

Detecting and quantifying the endogenous peptides in urine, which span a wide concentration range, has posed a challenge for a single technique. Mullen et al. couple a reflectron time-of-flight analyzer with a capillary electrophoresis (CE) system or a nanoflow high-performance liquid chromatography (HPLC) to generate a catalog of ~ 4500 urinary peptides [138]. Despite the challenges of low peptide abundance and high concentrations of salts and metabolites in urine, a study by Yang et al. used highly ordered mesoporous silica particles to extract peptides [139]. A total of 193 peptides were identified many of which were derived from proteins not commonly found in urine proteome databases. Urinary peptidomics has also been applied to assess age-related peptidome changes in healthy and diseased individuals ranging from 20 to 86 years old [140]. A peptidomic analysis of urinary exosomes led to identification of 3115 unique endogenous peptide fragments corresponding to 942 proteins [141]. A comprehensive analysis of urine proteome and peptidome was performed by Di Meo, et al. using an integrated analytical protocol developed with ultrafiltration [142]. The analysis of urine samples from healthy individuals led to the identification of 1754 proteins in addition to 4543 endogenous peptides derived from 566 proteins in the peptidomic analysis [142]. More recently, a comparative peptidomic analysis of plasma and urine was carried out to explore the origin of peptides in urine. This study identified 561 plasma and 1461 urinary endogenous peptides with only 90 peptides detected in both urine and plasma suggesting that most plasma peptides are not present in urine potentially due to tubular reabsorption [41]. In addition to the above-mentioned efforts, several other peptidomic studies have also been conducted in past decade [143–146].

### Urine peptidomics for biomarkers in renal cancer

The field of urinary peptidomics has shown great promise in identifying biomarkers linked to renal cancer, in particular renal cell carcinoma (RCC). RCC is asymptomatic at early stage and at the time of clinical presentation, the tumor has frequently progressed to an advanced stage [147, 148]. This emphasizes the necessity for early detection and accurate diagnostic methods [149]. A peptidomic analysis was conducted to explore urine peptide signatures that could differentiate malignant kidney tumors from benign masses and controls [150]. Using a MALDI-TOF profiling approach with urine pre-purification, two distinct peptide clusters were identified with higher abundance in patients’ urine and could be linked to proteins associated with tumorigenesis and progression, including meprin 1α (MEP1A), probable G-protein coupled receptor 162 (GPCR162), osteopontin (OPN), phosphorylase b kinase regulatory subunit alpha (PHKA1) and secreted and transmembrane protein 1 (SECTM1) [150]. This study demonstrated the potential of urinary peptidomics in detecting peptide-based biosignatures that can distinguish malignant RCC from benign tumors and healthy controls with potential applications for biomarker discovery for better outcome and disease management. An attempt of urinary peptidomics for discovering a peptide-based biomarker for progression and aggressiveness of RCC resulted in identification of numerous urine peptides in correlation with grading and staging of tumor [151]. Using MALDI-TOF profiling, this study identified 15, 26 and 5 peptides in urine which showed significant alteration of their urinary concentration in concordance with tumour size, stage and grade, respectively [151]. With the potential to enhance early diagnosis and treatment approaches, these findings offer a strong foundation for future studies to evaluate the usefulness of these peptides as possible biomarkers for managing RCC patients. Early detection and prognostic evaluation of RCC are critical for effective management, especially in cases of small renal masses (SRMs) [152]. A quantitative peptidomic approach was deployed to identify potential diagnostic and prognostic biomarkers for early-stage RCC-SRMs revealed the 9 endogenous urinary peptides significantly overexpressed in RCC-SRM [153]. Furthermore, two urinary peptides showed significantly higher expression in progressive clear cell RCC-SRMs compared to nonprogressive cases highlighting the utility of peptide biomarker in escalating clinical diagnosis and disease management [153]. These works illustrate the application of urinary peptidomics in renal cancer research and highlight the potential benefits for better RCC diagnosis and treatment.

### Urine peptidomics in chronic kidney diseases (CKD)

Urine peptidomics has been exploited in non-cancer disorders to delve into possible biomarkers and molecular signatures connected to different ailments. This covers a broad spectrum including chronic kidney disease (CKD) [42], cardiovascular diseases [154], diabetes [137, 155], autoimmune disorders, infectious diseases [156] and neurological conditions [157, 158]. The urinary peptidome is primarily derived from the filtration and reabsorption processes within the kidneys. As CKD is a condition characterized by impaired kidney function, studying urinary peptides provides valuable insights into kidney health and dysfunction. A CE-MS-based peptidomic study of urine was conducted to generate a database consisting of 5,010 unique urinary peptides presenting a diverse pool of potential biomarkers for diagnosing and monitoring various diseases including CKD [42]. Another study aimed at screening a high-risk population for developing CKD utilized CE-MS-based urinary peptidomics for identifying a new classifier i.e., CKD273 classifier attributed to different stages of CKD for early risk assessment and intervention [159]. Around 5000 urinary peptides were identified in a mass spectrometry analysis from apparently healthy individuals as well as patients with CKD of which 63 collagen-derived peptides were significantly upregulated and found to be associated with glomerular filtration rate (GFR) [160]. These results point to a substantial correlation between kidney function loss and collagen peptides suggesting that decreased collagen breakdown rather than increased production may be the cause of fibrosis in other organs as well [160]. In 2022, a peptidomic analysis was performed to compare different mass spectrometry approaches including CE-MS, LC–MS and MALDI-MS for profiling samples from diabetic nephropathy patients [161]. The study concluded that a front-end separation plays a crucial role in reliable peptide identification at the MS level providing a better technological approach to be deployed for urine peptidomics [161]. Recently, a link between glycosylation and CKD was established using CE-MS-based urine peptidome analysis which reflected 17 O-linked glycopeptides primarily derived from insulin-like growth factor-II (IGF2). One of these 17 glycopeptides showed a strong negative correlation with age-related estimated glomerular filtration rate (eGFR) suggesting its utility as a peptide biosignature in diagnosis of CKD [162]. In summary, urine-based peptidomics presents a promising approach for better understanding the molecular mechanisms driving the course of CKD and for identifying new biomarkers for early diagnosis and prognosis.

## Promises and pitfalls

The field of urinary proteomics holds promise for identifying biomarkers using non-invasive sampling techniques [4, 163]. Understanding the mechanisms behind disease can be greatly enhanced by analyzing the urine proteome not only for kidney-related disorders but also for the conditions affecting distant organs [66, 163–165]. Nonetheless, proteomic analysis of urine presents unique challenges such as variability [166, 167] including variation in protein abundance and contaminants [168, 169]. Thus, an appropriate proteomics experiment involving urine necessitates standardized procedures to minimize such variables. It is also important to note that the path from discovery of molecular biomarkers to clinical translation can be quite arduous and not always successful. Studies towards biomarker discovery efforts are best begun with a clear clinical question accompanied by a sound experimental design. Reliable identification and validation of biomarkers can be facilitated by high-throughput and sensitive platforms. Despite all these limitations, urinary proteomics shows significant promise in the development of non-invasive diagnostics eliminating the need for invasive procedures and offering a more patient-friendly approach to healthcare [163, 164]. Box 1 summarizes the different aspects for urinary proteomics including applications and challenges in the field.

## Conclusions

MS-based analysis of urinary proteins as well as post-translational modifications such as glycosylation has led to cataloging of a number of proteins originating from both urinary tract and distant organs. Promising biomarkers have been identified in urine as well as extracellular vesicles harvested from urine. Research in this area has evolved significantly through the use of analytical technologies such as nanoflow LC–MS/MS and could be further enhanced with newer methods like ion mobility spectrometry. Overall, such developments in characterizing the urinary proteome could have a high impact on how diseases are diagnosed and treated non-invasively in the future**.**

Box 1: Summary of urinary proteomics: considerations for sample preparation, applications and challenges Considerations:

- Selection of appropriate urine collection method: First-morning, 24-h collection or random (spot) urine collection
- Sample processing: Removal of cell debris, bacterial cells, salts
- Sample storage: Avoiding time at room temperature after collection; freezing at – 20 ℃
- Normalization of urine proteins: Normalization with urine creatinine

Applications:

- Non-invasive biomarker discovery: Detection of urinary proteins that can diagnose diseases arising from kidney (e.g., chronic kidney disease) as well as non-kidney related disorders (e.g., cancer)
- Monitoring of drug toxicity: Assessment of drug-induced toxicity by analyzing changes in the urinary proteome
- Point-of-care testing: Testing for specific proteins in a clinical setting

Challenges:

- Inter and intra-individual variability
- Variation in protein abundance
- Normalization of proteins
- Interfering compounds
- Standardization of analytical approaches

## Data Availability

No datasets were generated or analysed during the current study.
